# Optimizing basal body temperature measurement for cycle diagnostics: a comparison of different methods in female recreational athletes

**DOI:** 10.3389/fspor.2025.1732233

**Published:** 2026-01-16

**Authors:** Jana Nolte, Malina Pilz, Kirsten Legerlotz, Petra Platen

**Affiliations:** 1 Department of Sports Medicine and Sports Nutrition, Faculty of Sport Science, Ruhr University Bochum, Bochum, Germany; 2 Department of Movement and Training Sciences, Institute of Sport Sciences, University of Wuppertal, Wuppertal, Germany

**Keywords:** core body temperature, cycle tracking, intravaginal sensor, menstrual cycle, ovulation, temperature shift

## Abstract

**Introduction:**

Accurate detection of ovulation is essential for menstrual cycle-based training periodization for female athletes. Although body temperature tracking is a non-invasive method for this purpose, its reliability can vary depending on the measurement site and timing. This study aimed to compare the utility of different body temperature measurement methods and measurement times for ovulation detection.

**Methods:**

Seventeen recreationally active women tracked one menstrual cycle (October 2023 to February 2024) using continuous intravaginal core body temperature measurement as the reference method. Additional temperature measurements (sublingual, rectal and external ear) were taken at two points in time: 6 a.m. and upon waking. Ovulation was additionally confirmed using luteinizing hormone urinary tests. The post-ovulatory temperature increase was analyzed using both the validated Vollman method and the practical “three over six” rule for the four different methods and two different time points. Agreement of the measurement methods and times was assessed via Bland–Altman plots and concordance correlation coefficients.

**Results:**

The continuous intravaginal temperature sensor consistently identified ovulation with acceptable accuracy, detecting all ovulations and showing the largest temperature rise from follicular to luteal phase (0.31 ± 0.18 °C). Sublingual 6 a.m. measurements showed smaller temperature increases (0.17 ± 0.24 °C) and moderate agreement with the reference, whereas rectal (0.12 ± 0.21 °C) and external ear temperatures (0.22 ± 0.38 °C) exhibited higher variability and limited reliability. One cycle was confirmed as anovulatory and correctly identified by the intravaginal sensor.

**Discussion:**

The most reliable method for detecting ovulation in physically active women is continuous intravaginal temperature measurement. If this method is not available, a trained sublingual temperature measurement at a fixed time can be an acceptable, cost-effective alternative. All methods have their limitations.

## 1 Introduction

As athletic training and the conditions surrounding competitive sports can negatively affect an athlete's menstrual cycle (MC) and as hormonal variations throughout the MC may impact performance and training adaptations (1–4), precise diagnostics of the various phases of the MC are crucial for monitoring cycle health and implementing cycle-oriented and /or cycle-based training periodization approaches, as well as for conducting scientific studies involving women (4–6).

The gold standard for MC diagnostics is serial intravaginal ultrasound examination of the ovaries, a procedure that must be carried out by gynecologists in a clinical setting. This method allows direct observation of follicle development and ovulation (7, 8). In the field of sports science research, the combination of three methods is recommended for the diagnosis of the MC: calendar-based counting method, ovulation prediction using luteinizing hormone (LH) urine sticks and hormone analysis in venous blood (5, 8–11). MC monitoring requires reliable, non-invasive, cost-effective and simple methods that are suitable for everyday use to document ovulation characteristics. While recording the bleeding history is relatively simple, determining ovulation and the integrity of the associated cycle phases is a greater challenge. A frequently used valid and reliable method to detect ovulation is the measurement of the LH rise by application of antibody coated sticks in the morning urine (12). However, urinary LH testing may show variability in duration, intensity, and reliability, particularly in athletes (12–14).

An alternative non-invasive measurement method suitable for detecting ovulation is the basal temperature (BT) measurement (8). BT is defined as the lowest physiological body temperature attained at rest, typically occurring during sleep (15). For practical reasons, temperature is most commonly measured immediately upon waking, at which point it is referred to as the wake-up temperature. It serves as an indirect indicator of ovulation due to the progesterone-induced temperature rise of at least 0.2–0.5 °C during the luteal phase (LP) (8). The detailed metrics of the BT profile along the MC are as follows: During the follicular phase (FP), BT remains in the lower range (36.1–36.7 °C) until approximately one day before ovulation, when it may reach its lowest point (nadir) (12, 16, 17). However, the nadir is not considered a reliable indicator of ovulation (18). During the LP, the ruptured follicle, now referred to as the corpus luteum, begins to synthesize and release progesterone (19). As a result, the body temperature rises and forms a plateau. As the corpus luteum regresses and progesterone levels drop, BT returns to its baseline level within one to two days, coinciding with the onset of menstrual bleeding. This biphasic pattern of BT serves as a reliable retrospective indicator of the occurrence of ovulation (12, 20, 21), even if it does not indicate the exact day of ovulation. The results of BT monitoring and their interpretation can be affected by several factors. These include variations in measurement times, thermometer accuracy and the use of subjective judgment in interpreting questionable temperature fluctuations (8). To use BT for MC diagnostics, sufficient knowledge of the possible confounding factors that influence body temperature measures, such as fever, wake-up time and general measurement errors, is required (9, 18). The greatest challenge here appears to be the intra-individual and inter-individual variation in waking and sleeping times (8). While peripheral body temperature can be measured in the morning using ordinary thermometers (infrared, stick) (22, 23), temperature sensors have recently been developed to overcome the challenges of measurement and to optimize the use of BT in cycle diagnostics. Modern intravaginal temperature sensors can continuously determine the current core body temperature (CBT) value over 24 h, considering diurnal variations in temperature profiles (24). These systems record CBT at short intervals across the entire menstrual cycle and employ algorithm-based analyses to identify the fertile window. Previous work using such vaginal biosensors demonstrated reliable retrospective ovulation detection and prediction of fertile days in the general population (24, 25). However, these studies focused primarily on fertility awareness and did not compare intravaginal CBT with other temperature measurement sites or fixed measurement times, nor did they investigate physically active or athletic women.

The present study therefore extends this literature by directly comparing three different BT measurement methods (external ear canal, sublingual, and rectal methods) and two timings (6 a.m., and the individual wake-up time) against continuous intravaginal CBT in recreational athletes. The goal was to determine which method is most suitable for monitoring menstrual cycle phases in athletic context (recreational). We hypothesized that continuous intravaginal temperature monitoring provides the most accurate and physiologically meaningful temperature profiles, allowing reliable retrospective identification of ovulation and corpus luteum sufficiency. We further assumed that single-point measurement methods, such as sublingual, ear canal or rectal recordings, would show limitations in accuracy and consistency, particularly for women engaging in athletic activity.

## 2 Materials and methods

### 2.1 Participants

An *a priori* sample size calculation for the agreement analyses between temperature measurement methods was performed based on the Bland–Altman approach as described by Lu et al. (26). Assuming a standard deviation of the differences of 0.14 °C (combined measurement error), pre-specified limits of agreement of ±0.30 °C, an alpha level of 0.05 and a statistical power of 80%, the required sample size was 102 measurement points, corresponding to at least five menstrual cycles with one observation per method.

In the present study, 17 recreationally trained women (age: 24 ± 2 years; gynecological age: 10 ± 2 years; height: 169.7 ± 5.6 cm; body mass: 65.4 ± 6.0 kg) from different disciplines (Tier 2) were recruited (27). Participants trained at least 6 h per week and were involved in track and field (*n* = 4), endurance sports (*n* = 4), team sports (*n* = 7), and individual sports (*n* = 2), had a regular sleep behaviour (e.g., no shift work), had not used hormonal contraception in the previous 12 months and during the intervention period and had a regular MC (defined as at least nine cycles between 21 and 35 days per calendar year during the previous year (mean past cycle length: 28 ± 2). All participants received detailed information about the study procedures and gave written informed consent for their participation. The study was conducted in accordance with the tenets of the Declaration of Helsinki and the protocol was approved by the local ethics committee of the Faculty of Sport Science, Ruhr University Bochum (ethics vote: EKS V 2022_18).

### 2.2 Experimental design and procedure

A total of 17 women completed one full monitored menstrual cycle each, resulting in 17 cycles for analysis. In total, four temperature measurement methods were compared: continuous intravaginal core body temperature (reference standard), sublingual temperature, rectal temperature, and external ear canal temperature. The continuous intravaginal core body temperature measurement served as the reference standard for all comparisons.

Data were collected over the course of a complete MC, beginning on the first day of each cycle. The calendar-based method was used to determine cycle and bleeding length. Data on CBT were collected only once bleeding had ceased, from day six of the MC at the latest. All measurements were self-administered by the participants. CBT was measured continuously using an intravaginal temperature sensor. The other temperature measurements were done every morning at two or three different sites [external ear canal, sublingual, and, on a voluntary basis, also rectal (*n* = 3)] and at two different times: at 6 a.m. and immediately after “normal” waking. The participants used an infrared ear thermometer and two digital stick thermometers for the external ear canal, sublingual and rectal measurements, respectively. Temperature measurement started with the ear canal, followed by the sublingual and rectal measurements. All measurements were taken twice in succession. If there was a deviation of 0.2 °C between the first and second measurement at each respective site, a third measurement was taken. The first spontaneous morning urine was collected from day six of the cycle until ovulation and the sample was analyzed directly for LH to detect a preovulatory LH surge.

### 2.3 Measurements

These measurements methods were selected because they represent the most commonly used and readily accessible approaches and already have a proven scientific background (23, 24).

#### 2.3.1 Core body temperature (CBT)

CBT was continuously measured using an intravaginally worn temperature sensor (OvulaRing, VivoSensMedical GmbH, Leipzig, Germany). The OvulaRing is a certified medical device. The accuracy of the biosensor is reported to be ±0.1 °C. For validation, a water bath test was performed at ©inotec FEGmbH with reference to an externally calibrated thermometer (OVU TD DOC 61403-1 report on measurement accuracy, Alexander, 2022). The sensor is embedded in a flexible plastic ring and can be worn effortlessly for almost an entire MC, usually except during menstrual bleeding. It can be removed and re-inserted at any time, if necessary, or if the participants feel uncomfortable. The sensor measures and stores data every 5 min, generating 288 temperature readings over 24 h. The data can be read via Bluetooth after the sensor is removed from the vagina. A 24-h CBT profile is generated from the data and analyzed by the associated application. CBT at any time can be extracted from the raw data. In our study, the sensor was inserted into the vagina after the end of menstruation or no later than day 6 of the cycle and removed on the first day of the next period.

#### 2.3.2 Temperature in the external ear canal

External ear canal temperature (ear temperature) was measured using a commercially available infrared ear thermometer (IRT6520MNLA, Braun, Bussigny, Switzerland). The accuracy of the thermometer is reported to be ±0.2 °C (23). Participants were individually instructed on how to use the thermometer to obtain accurate data.

#### 2.3.3 Sublingual and rectal temperature

Sublingual and rectal (optional) temperature measurements were performed using a conventional rod thermometer (PRT2000EU, Braun, Bussigny, Switzerland). The accuracy of this thermometer is reported to be ±0.1 °C (23). Sublingual temperature was measured under the tongue with the mouth closed and rectal temperature was measured intrarectally, each for approximately 10 s. Participants were individually instructed on how to use the thermometer to obtain accurate data.

#### 2.3.4 Urinary analysis of LH

Spontaneous early morning urine was semi-quantitatively tested for increases in the hormones estradiol (E2) and LH between menstruation and ovulation according to the manufacturer's instructions (Clearblue® Ovulation Test Advanced & Digital, Swiss Precision Diagnostics GmbH, Geneva, Switzerland). The sensitivity of the LH detection of the Clearblue® Ovulation Test Advanced & Digital is 40 mIU/mL when measured according to the 3rd International Standard for a urinary LH and FSH for Bioassay (71/264). The results of the E2 analysis were not used in this study. The days on which a positive test result for LH was obtained were noted.

### 2.4 Data processing

The lowest night-time temperature recorded by the continuous intravaginal temperature sensor was defined as BT (Intravaginal basal). The temperature at exactly 6 o'clock (Intravaginal 6am) and the temperature after normal waking (Intravaginal wake-up) were identified from the 24-h data. The other measurement methods were also filtered for a 6 a.m. measure and wake-up measure by averaging the two closest measurements from the respective temperature recordings at the various measurement times.

Date of ovulation was defined as LH peak plus one day (5). Additionally, ovulation day from the 24-h intravaginal recordings was identified using the automated detection algorithm provided by VivoSensMedical GmbH within the OvulaRing application. This was then confirmed by visual inspection of the temperature profiles and LP temperature rises by three independent reviewers. When the automated OvulaRing algorithm did not provide an ovulation estimate, manual adjudication was performed by the company's specialist team and the authors using established temperature-charting criteria. Specifically, ovulation was identified when the 24-h CBT profile showed a sustained rise above the mean of the preceding days (“crossing-the-line” rule), consistent with the classical three-over-six principle. All cases were reviewed jointly, and consensus was reached for each cycle.

To compare the date of ovulation (LH peak plus one) with the other temperature methods, an additional analytical approach was applied. First of all, the validated Vollman method was used to identify a temperature shift in each method and time point (21, 28). In this method, the cycle-mean temperature is calculated from all measurements, and the point where the temperature curve crosses this mean is taken as the dividing line between the FP and LP (21). For cycles in which the intravaginal sensor was inserted after menstruation, the first six days of missing CBT data were imputed by linear interpolation between the last available value before insertion and the first valid continuous measurement. This conservative approach assumes a stable low-temperature profile during the early follicular phase and avoids introducing artificial fluctuations. The imputed segment accounted for a maximum of six days per cycle (≤21% of all time points). All analyses were repeated with and without imputation. Secondly, the practical implemented “three-over-six” rule, which is defined as the first day on which three consecutive temperatures are at least 0.2 °C higher than the preceding six, was used (29–31). These rules were applied to all measurement methods at the defined time points. The results were summarised based on the number of detected temperature shifts and the deviation in days from the respective ovulation day. This enabled a comparison of the detection rates and temporal accuracy.

Cycles in which neither a temperature rise nor a positive LH test was observed were classified as anovulatory and excluded from further analysis.

### 2.5 Statistical analysis

In a first step, parameters were assessed at an individual level and presented descriptively. For the purpose of group analysis, the data were allocated to either the FP or the LP, with ovulation day serving as the dividing line.

In a second step, external ear, sublingual and rectal temperatures at 6 a.m. and after awakening were compared with intravaginal temperatures at the respective times. Normality was assessed using the Shapiro–Wilk test applied to the pairwise differences, as required for Bland–Altman analysis. Some differences deviated from normality, particularly for ear and sublingual temperatures, which is expected for physiological temperature measurements. No transformations were applied, as Bland–Altman analyses are robust to moderate deviations from normality and temperature values cannot be meaningfully interpreted on a transformed scale. The comparisons were made using Bland-Altman plots. For statistical analysis, the intraclass correlation coefficient [ICC(2,1)] was used to assess the agreement between the methods. The use of Bland-Altman plots and ICC(2,1)s allowed for detailed analysis of the agreement and systematic differences between methods. Data are presented as mean ± standard deviation.

## 3 Results

### 3.1 24-h temperature profile

**Figure 1:**
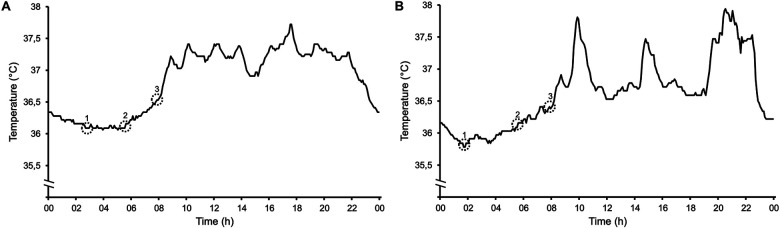
Exemplary 24-h CBT profiles of two subjects **(A,B)**. Numbers indicating (1) minimum temperature plateau during the night; (2) temperature at 6 a.m.; (3) temperature upon awakening.

Representative 24-h temperature profiles of one day from two participants (A and B) are presented to illustrate the physiological basis for differences between different measurement times (see Figure 1). In example A, a distinct temperature minimum was reached around 03:10 a.m., forming a plateau that persisted until approximately 06:00 a.m., when the participant was awakened. After returning to sleep, CBT gradually increased from 36.1 °C to 36.6 °C when the participant spontaneously woke up at 08:00 a.m. and further to 36.8 °C within 15 min post-awakening. A distinct temperature rise was observed immediately following the wake-up stimulus. In example B, the lowest temperature was recorded at 01:40 a.m., at 35.8 °C. By 6:00 a.m., it had risen to 36.2 °C, increasing further to 36.4 °C at 08:00 a.m. upon awakening. Within this participant, exercise-induced increments in CBT are clearly detectable at approximately 10 a.m., 3 p.m. and 8 p.m.

### 3.2 Cycle characteristics and phase determination

17 MCs were included in the analysis. The average MC duration was 28.5 ± 3.8 days, and ovulation occurred on average on day 17.4 ± 3.4, while the urinary LH test was positive on average on day 16.8 ± 3.4. This resulted in an average LP length of 11.3 ± 2.6 days based on LH data. In five MCs, the OvulaRing algorithm failed to detect ovulation automatically (e.g., according to short LP or single temperature fluctuations around ovulation). Visual diagnosis of the temperature profiles could detect ovulation in another four cycles. Unique MC characteristics were observed in four cycles. Two cycles had a below-average LP length of 5 and 6 days. One cycle was documented with a cycle length of 39 days and an above-average FP length of 25 days. One cycle was diagnosed as anovulatory.

The average temperature increases from FP to LP showed the highest average values and the lowest scatter in the measurements *Intravaginal basal* (0.31 ± 0.18 °C), *Intravaginal 6am* (0.34 ± 0.21 °C) and *Intravaginal wake-up* (0.36 ± 0.21 °C). The *ear 6am* temperature rose less from FP to LP compared to the intravaginal measurements and varied the most of all measurements (0.22 ± 0.38 °C). *Sublingual 6am* temperature rose even less with a medium variation (0.17 ± 0.24 °C) and *rectal 6am* temperature rose the least with a medium variance (0.12 ± 0.21 °C). The values at spontaneous awakening (08:06 a.m. ± 01:08 h) were consistently higher than those at 6 a.m.

According to the Vollman method and the “three-over-six” rule, temperature rise was detected in all 16 biphasic cycles from CBT (*Intravaginal basal*) data, with an average deviation of three days (Vollman method) two days (3-over-six) from the LH peak, as described below (Table 1). The other measurement methods showed a lower detection rate and larger deviations compared to the actual ovulation day (Supplementary Tables A1–A3). The conclusions with imputation in the Vollmann method (*Intravaginal*) did not change compared to the results without imputation.

**Table 1:** Descriptive data for comparison of ovulation detection methods by urine LH peak and the temperature shift by two different quantitative basal temperature methods using intravaginal temperature measurement at night (intravaginal basal).

Analytical method	Cycles (n)	Cycle length (days)	Shift day (day)
LH peak	16	28.5 ± 3.8	16.8 ± 3.4
Vollman method	16	28.5 ± 3.8	19.8 ± 3.1
3-over-6 rule	16	28.5 ± 3.8	18.8 ± 3.3

All data are mean ± SD.

### 3.3 Agreement between the temperature measurement methods

**Figure 2:**
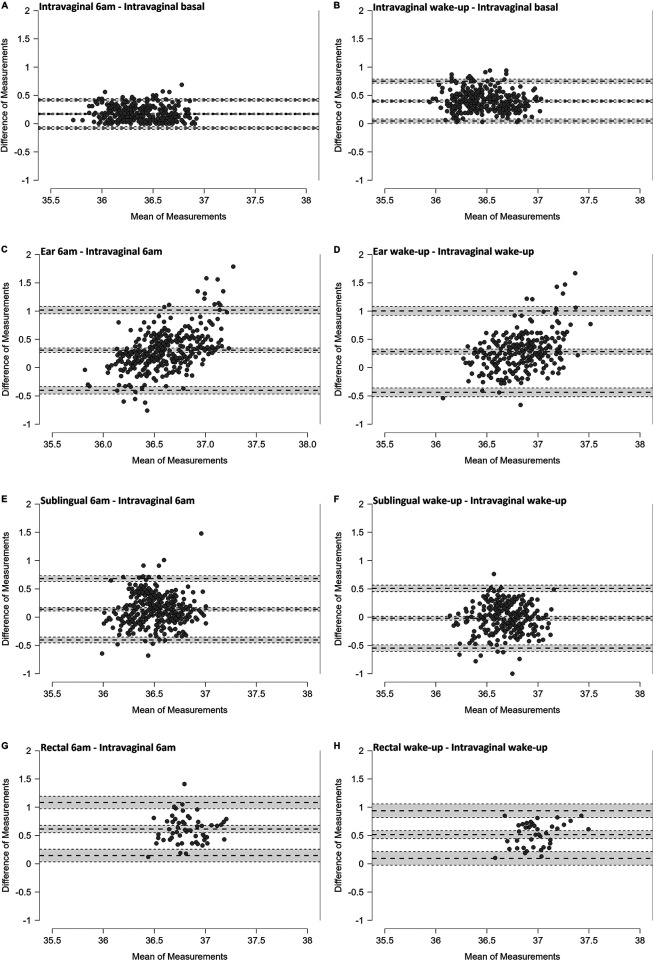
Bland–Altman plots illustrating the deviation of the intravaginal temperatures at 6 a.m. and upon awakening from the intravaginal basal temperature **(A,B)** and the deviation of the temperatures obtained with the other methods from the respective intravaginal measurements at 6 a.m. and upon awakening, respectively **(C–H)**. The dashed lines represent the mean difference (MD) and the upper and lower limits (LoA). The shading indicates the 95% confidence interval. The number of included data points were: **(A)** intravaginal 6am (*n* = 375); **(B)** intravaginal wake-up (*n* = 305); **(C)** ear 6 am (*n* = 360); **(D)** ear wake-up (*n* = 269); **(E)** sublingual 6am (*n* = 360); **(F)** sublingual wake-up (*n* = 269); **(G)** rectal 6am (*n* = 58); **(H)** rectal wake-up (*n* = 40).

Figure 2 shows Bland–Altman plots that compare the temperatures obtained using each measurement method with the BT [sublingual (*n* = 17), ear (*n* = 17), rectal (*n* = 3, only exploratory)] from the continuous intravaginal measurement, or the temperature from the continuous measurement at the respective time, respectively. The differences between each method and the respective reference measure are plotted against the mean of the two values. Mean differences (MD) and limits of agreement (LoA) for each method and time point are summarised in Table 2. Data from 17 participants were included in the analysis; three of them also provided rectal temperature data.

**Table 2:** Mean differences (MD) and limits of agreement (LoA) between data obtained from the different temperature measurements and the respective intravaginal data (*basal, 6am, upon awakening*).

Measurement	Reference	Upper LoA	MD	Lower LoA	ICC(2,1)	
		°C	°C	°C	PE	95% CI
*Intravaginal 6am*	*Intravaginal basal*	0.42	0.17	−0.08	0.69	[0.03, 0.87]
*Intravaginal wake-up*	*Intravaginal basal*	0.75	0.40	0.05	0.33	[−0.09, 0.67]
*Intravaginal wake-up*	*Intravaginal 6am*	0.55	0.23	−0.10	0.6	[−0.05, 0.83]
*Ear 6am*	*Intravaginal 6am*	**1**.**02**	**0**.**31**	**−0**.**40**	0.31	[0.05, 0.50]
*Sublingual 6am*	*Intravaginal 6am*	**0**.**68**	**0**.**14**	**−0**.**40**	0.32	[0.20, 0.43]
*Rectal 6am*	*Intravaginal 6am*	**1**.**08**	**0**.**61**	**0**.**15**	0.08	[−0.06, 0.26]
*Ear wake-up*	*Intravaginal wake-up*	**1**.**00**	**0**.**28**	**−0**.**44**	0.25	[0.02, 0.43]
*Sublingual wake-up*	*Intravaginal wake-up*	**0**.**51**	**−0**.**02**	**−0**.**55**	0.42	[0.34, 0.49]
*Rectal wake-up*	*Intravaginal wake-up*	**0**.**94**	**0**.**52**	**0**.**09**	0.21	[−0.07, 0.53]

Values exceeding the ±0.5 °C reference threshold are highlighted in the table (bold text).

On average, the lowest temperature occurred at 03:24 a.m., with individual values ranging from 09:50 p.m. to 07:00 a.m. The *Intravaginal 6am* temperature was slightly and consistently higher than the basal temperature, with small LoA. The *Intravaginal wake-up* temperature was clearly higher than the basal temperature, with small to medium LoA.

Of all the other methods, sublingual temperatures recorded at 6 a.m. differed the least from the intravaginal reference. At this time, there was the greatest difference in rectal temperatures and the LoA was highest for ear temperatures. At the wake-up time point, sublingual temperatures again showed the smallest MD, although there was a wide range of individual variations. Ear and rectal wake-up temperatures exhibited greater deviations from the reference, with wider LoA in both directions.

The correlation between the temperatures obtained using each of these methods (ear, sublingual and rectal) and the respective intravaginal reference temperature was weak in all cases [ICC(2,1) < 0.4].

## 4 Discussion

This study provides important insights into the application of temperature-based MC diagnostics in sports practice. As hypothesized, 24-h intravaginal CBT monitoring proved sufficiently accurate and reliable for identifying ovulation in recreational athletes during habitual training, whereas other measurement methods and fixed measurement times showed clear limitations. To our knowledge, this is the first study to directly compare multiple temperature measurement sites and standardized measurement times against continuous intravaginal CBT in recreationally trained women, providing a novel perspective on the practical applicability of these methods in a sports context.

A biphasic temperature pattern was automatically detected by the device algorithm in 12 out of 17 cycles. In five cycles, the algorithm did not classify ovulation, not because the temperature sensor failed, but because the algorithm applies strict criteria (e.g., minimum required luteal phase length, suppression of single-day fluctuations). Importantly, in four of these five cases, the biphasic pattern was clearly visible in the raw 24-h CBT data, allowing ovulation to be identified retrospectively. Only one cycle showed no temperature rise at all, indicating an anovulatory cycle (confirmed with missing LH surge).

In this study, the continuous 24-h intravaginal CBT measurement was used as the reference method against which all other temperature measurement methods were compared. The absence of ultrasonography does not affect the validity of the comparison between temperature-based methods, as no absolute ovulation date was required for evaluating agreement between temperature measures (32). Although it is not possible to determine an exact ovulation day using evaluation methods such as the Vollman method and the “three over six” rule, it is at least possible to assess the function of the corpus luteum and thus confirm the occurrence and estimate the time of ovulation (28, 31, 32). The findings of this study therefore underscore the efficacy of nocturnal BT determination as an indirect diagnostic tool for tracking ovulation based on the evidence of a functioning corpus luteum in female athletes.

The rationale for employing body temperature measurement in general cycle diagnostics and specifically for the detection of ovulation, is predicated on the fact that, following ovulation, the corpus luteum produces progesterone in sufficient quantities (32, 33). Progesterone has a thermogenic effect, which Baker et al. attribute to endocrine-induced changes in the thermoregulatory set point. This effect is further explained at the neurophysiological level by Rai et al., who suggest that progesterone acts on the preoptic area of the hypothalamus by inhibiting heat-sensitive and activating cold-sensitive neurons, thereby raising the temperatures at which sweating, cutaneous vasodilation, thermal comfort and chilly feelings begin. As a result, CBT increases (34). In cases of corpus luteum insufficiency, a non-detectable or only marginal rise in progesterone, reflected by a temperature increase of less than the defined threshold of 0.2 °C, would therefore be classified as an anovulatory cycle (31). The temperature rise occurs approximately 1–3 days after the peak LH surge, but with considerable interindividual variation (28, 35). Early studies have even indicated a deviation of up to four days (19). Nevertheless, the BT measurement method advocated by Lui et al. is considered as promising method for continuous, longitudinal monitoring of ovarian function.

The ovulation day was utilized as the diagnostic reference point in this study, based on the expected post-ovulatory body temperature increase ranging from 0.2 °C to 0.5 °C and under the assumption of adequate corpus luteum function (8). Consequently, any measurement method employed for the detection of this rise must demonstrate a variability not exceeding 0.5 °C, which signifies a clinically meaningful threshold.

The variability of temperature measurements depending on the time of recording is attributable to physiological factors. Body temperature exhibits diurnal fluctuations, reaching its nadir during nocturnal sleep (36). The precise timing of this nocturnal nadir varies both intra- and inter-individually and is influenced, among other factors, by sleep patterns (36, 37). The findings of this study demonstrate unequivocally that CBT varies physiologically over a 24-h period and that the timing of the nocturnal minimum differs considerably among athletes. This 24-h temperature pattern underscores the impact of the circadian rhythm and external factors (e.g., wake-up time, physical activity) on body temperature. The continuous intravaginal temperature sensor's capacity to accurately capture the true nocturnal nadir is a significant advantage over conventional methods of temperature monitoring, such as fixed-time recordings (e.g., at 06:00). These methods may fail to detect or accurately record the true minimum temperature.

The utilisation of alternative temperature measurement methods, in conjunction with the variability in measurement timing gave rise to notable deviations from the intravaginal data. None of these methods proved to be reliable. Utilising commercially available clinical thermometers with the Vollman method, only 50% of the biphasic cycles were correctly identified, despite 100% being correctly confirmed as biphasic by the intravaginal methods. Furthermore, estimates of ovulation day deviated by up to 10 days when thermometers were used in comparison to the continuous intravaginal temperature sensor. This discrepancy can be partly attributed to measurement variability exceeding the threshold of 0.5 °C, which is defined as clinically meaningful. The limited measurement accuracy observed in this study is consistent with the findings of previous research on the use of body temperature for the purpose of ovulation and fertility diagnostics. It has been demonstrated by earlier studies that the accuracy of temperature-based methods is influenced by several factors. This includes the type of thermometer used, the measurement location and the time of measurement. In addition, external variables such as sleep duration, alcohol consumption, exposure to heat and cold and fever must be taken into account (12, 18, 35).

A number of studies have previously evaluated the accuracy of thermometers (23). These studies identified significant differences between devices and concluded that measurement data from the Braun infrared thermometer (IRT6520), which was used for temperature assessment in the external auditory canal in the present study, showed the best correlation with a gold standard (the SureTemp Plus medical thermometer from Welch-Allyn). However, in our study on indirect ovulation diagnostics using external ear canal temperature, we were unable to confirm a high level of accuracy in practical application. Theoretically, the configuration and the algorithmic processing of the infrared thermometer should guarantee that the temperature displayed is reflective of the tympanic membrane, as opposed to any other component of the ear canal. This is typically achieved by selecting the highest value from a series of measurements captured at a wide angle around the tip of the sensor (38). However, if the probe makes contact with the canal wall rather than aligning with the tympanic membrane, which is plausible due to anatomical variability, or if the measurement is affected by small hairs in the canal, reproducibility is compromised and the recorded temperature tends to be too low (39). In the present study, the Braun rod thermometer (PRT2000) was found to yield smaller average deviations from the intravaginal reference method when employed for sublingual measurements. It is widely accepted that sublingual measurements are expected to indicate lower temperatures compared to intravaginal measurements owing to the greater distance from the body's core (23). However, the sublingual temperature measured at 6 a.m. and immediately after awakening was, on average, virtually identical to the intravaginal temperature at the same time. This phenomenon is likely attributable to the absence of frequent mouth openings and the observation that, while remaining in bed, peripheral body temperature does not differ substantially from CBT or from intravaginal temperature, which is measured in close proximity to the core.

Continuous intravaginal CBT measurement is widely regarded as one of the most accurate non-invasive approaches for assessing core body temperature, yet several factors can introduce measurement variability. Because the sensor relies on indirect heat transfer from the vaginal epithelium, the recorded temperature may be influenced by local blood flow, tissue contact and thermal conductivity, and small positional shifts of the ring can lead to slight fluctuations in absolute values (25, 40). Although the OvulaRing has demonstrated high technical accuracy under controlled conditions, real-world factors such as physical activity, intermittent removal or changes in vaginal moisture may affect single-point measurements (OVU TD DOC 61403-1 report on measurement accuracy, Alexander, 2022). In addition, ovulation detection depends on the device's internal algorithm. Missed detections typically result from strict classification criteria rather than from shortcomings of the underlying CBT signal, which can still display a clear biphasic pattern (25, 41, 42). Overall, while intravaginal CBT is not a perfect measure of absolute temperature or ovulation timing, its continuous sampling and proximity to the body core make it one of the most robust non-invasive methods currently available for capturing the characteristic biphasic temperature shift.

In our study, we used LH measurements in urine for ovulation detection. In general, ovulation occurs 14–26 h after a rise in LH. The LH surge can vary significantly in both duration and intensity (14). While some women exhibit a rapid surge within a single day, others experience a gradual rise over several days (12). Moreover, an LH surge may be detected even in the absence of actual ovulation (13). Such “false-positive” results are particularly relevant in athletes, who may experience anovulatory cycles due to intense training or low energy availability (13). This variability complicates the accurate determination of the ovulation timing based on urinary LH testing. In our study, the time interval between the urinary LH surge and the subsequent rise in BT was 1.3 ± 3.3 days (max. 5 days; min. 0 days), which falls within the expected wide range of variation (12). The high standard deviation of this time interval is at least partly due to the variability of the configuration, amplitude and duration of the individual LH peaks (14). Therefore, the sole use of urinary LH measurements for ovulation detection seems questionable.

### 4.1 Limitations

This study has several limitations that should be acknowledged. First, ovulation was not confirmed using the gold standard of serial intravaginal ultrasonography, and serum progesterone concentrations were not measured due to resource constraints. Second, the relatively small sample size limits the generalizability of our findings, and the results should be interpreted as preliminary evidence. Third, potential external influences such as alcohol consumption, stress, or variations in sleep duration and quality, which are known to affect body temperature, were not systematically controlled. Finally, although training load was monitored, it was not included in the statistical analysis and may represent an additional confounding factor. Future research with larger cohorts and multimodal assessment, including hormonal markers and gold-standard imaging, is needed to further validate and extend these findings.

### 4.2 Practical applications

The findings of this study highlight the potential of continuous intravaginal core body temperature monitoring as a valuable tool in sports practice. This method allows for the reliable assessment of luteal function and the retrospective confirmation of ovulation. Such information can support the development of individualized, cycle-based training periodization approaches, helping athletes and coaches to better align training loads with hormonal phases of the menstrual cycle. In addition, continuous monitoring may assist in the early identification of menstrual disturbances, which are more prevalent among female athletes than in the general population (24, 43). Consistent with this, our study revealed one anovulatory cycle, two cycles with a shortened luteal phase, and one prolonged cycle. Nevertheless, intravaginal body temperature measurement proved robust even in these atypical cases, enabling reliable analysis despite such variations. Looking ahead, further sensor-based technologies such as smart rings, wristbands, and smartwatches may represent the future of menstrual cycle tracking after independent validation (35, 44). These devices, which are already gaining popularity in the healthcare market, may eventually replace conventional temperature-based methods by combining peripheral skin temperature measurement with artificial intelligence algorithms to provide accurate estimations of core body temperature.

## 5 Conclusion

This study demonstrates that commercially available thermometers used at defined peripheral body sites are not sufficiently reliable for identifying biphasic temperature patterns or accurately determining ovulation timing in female athletes. In contrast, continuous intravaginal monitoring of core body temperature proved to be the most accurate and robust method among those evaluated, providing reliable retrospective confirmation of ovulation and luteal function. As a more practical and cost-effective alternative, sublingual temperature measurement at a fixed time point may be considered, though with acknowledged limitations.

These findings underscore the value of accurate temperature-based menstrual cycle diagnostics in sports practice. Reliable identification of menstrual cycle phases can support a more individualized approach to menstrual health monitoring, enabling athletes and coaches to better adapt training and competition schedules to physiological changes across the cycle. Ultimately, this approach may contribute to optimizing both performance and long-term health in female athletes.

## Data Availability

The raw data supporting the conclusions of this article will be made available by the authors, without undue reservation.
