# Supplementary material for: Optimizing basal body temperature measurement for cycle diagnostics: a comparison of different methods in female recreational athletes
Source: Front Sports Act Living. 2026 Jan 16;7:1732233. doi: 10.3389/fspor.2025.1732233 (PMC12855485; doi:10.3389/fspor.2025.1732233)
Supplement: Supplementary file 1 [file Datasheet1.pdf]

Supplementary Material

1 Supplementary Data

Supplementary Table A1. Summary of detection rates for the Vollmann method and the “three over six” rule in all individual cases.

ID	LH	O	Ovula Ring	Method	Intrav. basal	Intrav. basal (imputation)	Intrav. 6am	Intrav. 6am (imputation)	Intrav. wake-up	Intrav. wake-up (imputation)	Sublingual 6am	Ear 6am	Rectal 6am	Sublingual wake-up	Ear wake-up	Rectal wake-up
BT_20	13	14	14	Vollman	16	16	16	16	16	16	14	failed	failed	failed	failed	failed
				3-over-6	15		15		failed		14	10	13	failed	failed	failed
BT_18	18	19	19	Vollman	20	20	21	20	20	20	failed	failed		failed	failed	
				3-over-6	19		20		failed		failed	21		failed	failed	
BT_17	25	26	26	Vollman	29	29	29	29	27	26	failed	28		failed	25	
				3-over-6	28		28		failed		28	28		28	24	
BT_16	17	18	16	Vollman	19	19	19	19	19	19	failed	20		18	18	
				3-over-6	18		18		18		11	failed		failed	failed	
BT_14	15	16	15	Vollman	17	17	17	17	16	16	17	17		18	18	
				3-over-6	16		18		17		failed	16		failed	failed	
BT_15	/	/	/	Vollman												
				3-over-6												
BT_13	18	19	19	Vollman	21	21	21	21	failed	failed	failed	failed		failed	failed	
				3-over-6	20		failed		16			16			9	
BT_12	16	17	19	Vollman	21	21	20	20	22	22	21	21		23	failed	
				3-over-6	20		19		23		failed	20		failed	15	
BT_11	14	15	14	Vollman	18	16	17	16	16	16	18	14		17	20	
				3-over-6	15		15		failed		17	13		19	19	

ID	LH	O	Ovula Ring	Method	Intrav. basal	Intrav. basal (imputation)	Intrav. 6am	Intrav. 6am (imputation)	Intrav. wake-up	Intrav. wake-up (imputation)	Sublingual 6am	Ear 6am	Rectal 6am	Sublingual wake-up	Ear wake-up	Rectal wake-up
BT_08	12	13	17	Vollman	18	18	18	18	17	17	17	18		17	failed	
				3-over-6	17		18		failed		failed	18		failed	failed	
BT_07	16	17	16	Vollman	18	18	18	18	18	18	17	19		18	20	
				3-over-6	17		17		17		1ß	16		failed	failed	
BT_06	10	11	14	Vollman	17	17	18	17	failed	failed	20	failed		20	failed	
				3-over-6	16		19		failed		failed	19		failed	failed	
BT_05	16	17	17	Vollman	19	19	19	18	14	14	16	15		14	14	
				3-over-6	23		18		13		failed	14		13	13	
BT_04	17	18	18	Vollman	21	20	21	21	20	20	failed	failed		failed	failed	
				3-over-6	19		failed		19		11	9		25	9	
BT_03	21	22	21	Vollman	24	24	24	24	25	25	failed	failed	failed	failed	failed	24
				3-over-6	22		23		failed		failed	13	failed	failed	failed	failed
BT_02	16	17	16	Vollman	18	18	19	19	19	19	failed	failed	failed			
				3-over-6	17		17		failed		failed	failed	failed			
BT_01	18	19	19	Vollman	20	20	20	20	20	20	19	failed		19	18	
				3-over-6	19		19		19		failed	failed		18	18	

Supplementary Table A2. Descriptive data for comparison of ovulation detection methods by urine LH peak and the temperature shift by two different quantitative basal temperature methods (Vollmann, 'three over six" rule) using intravaginal temperature measurement at night, 6 a.m. and after awakening. (All data are mean ± SD)

	LH	*Ovulation*	*OvulaRing*	*Intrav. basal*		*Intrav. 6am*		*Intrav. wake-up*		*Intrav. basal*	*Intrav. 6am*	*Intrav.*
				**Vollman**	**3-over-6**	**Vollman**	**3-over-6**	**Vollman**	**3-over-6**	**Vollman**	**Vollman**	**Vollman**
**n**	16	16	16	16	16	16	16	15	15	16	16	15
**MD ± SD**	16.8 ± 3.4	17.4 ± 3.4	17.5 ± 3.0	19.8 ± 3.1	18.8 ± 3.3	19.8 ± 3.0	18.9 ± 3.2	19.2 ± 3.5	17.8 ± 2.7	19.6 ± 3.2	19.6 ± 3.2	19.1 ± 3.3
**NoD**				16	16	16	14	14	8	16	16	14
**Deviation**				6	6	7	8	5	6	6	6	5
**Deviation (min)**				1	0	1	0	-3	-4	1	1	-3

MD: Mean differences
SD: Standard deviation
NoD: Number of detections
Deviation from ovulation day (LH plus one)

Supplementary Table A3. Descriptive data for comparison of ovulation detection methods by urine LH peak and the temperature shift by two different quantitative basal temperature methods (Vollmann, 'three over six" rule) using sublingual, ear and rectal temperature measurement at 6 a.m. and after awakening. (All data are mean ± SD)

	LH	*Ovulation*	*OvulaRing*	*Sublingual 6am*		*Ear 6am*		*Rectal 6am*		*Sublingual wake-up*		*Ear wake-up*		*Rectal wake-up*	
				**Vollman**	**3-over-6**	**Vollman**	**3-over-6**	**Vollman**	**3-over-6**	**Vollman**	**3-over-6**	**Vollman**	**3-over-6**	**Vollman**	**3-over-6**
**n**	16	16	16	16	16	16	16	3	3	15	15	15	15	2	2
**MD ± SD**	16.8 ± 3.4	17.4 ± 3.4	17.5 ± 3.0	17.7 ± 2.0	16.2 ± 6.3	19 ± 4.1	16.4 ± 4.8		13.0 ± 0.0	18.2 ± 2.3	20.6 ± 5.3	19.0 ± 3.1	15.3 ± 5.1	24 ± 0.0	
**NoD**				9	5	8	13		1	9	5	7	7	1	
**Deviation**				9	2	5	8		-1	9	7	5	4	2	
**Deviation**				-1	-7	-2	-9		-1	-3	-4	-3	-10	2	

MD: Mean differences
SD: Standard deviation
NoD: Number of detections
Deviation from ovulation day (LH plus one)
